# Depression genetic risk score is associated with anhedonia-related markers across units of analysis

**DOI:** 10.1038/s41398-019-0566-7

**Published:** 2019-09-19

**Authors:** Guia Guffanti, Poornima Kumar, Roee Admon, Michael T. Treadway, Mei H. Hall, Malavika Mehta, Samuel Douglas, Amanda R. Arulpragasam, Diego A. Pizzagalli

**Affiliations:** 1 Department of Psychiatry, Harvard Medical School, Boston, MA 02115 USA; 2 McLean Hospital, Belmont, MA 02478 USA; 3 Department of Psychology, University of Haifa, Haifa, Israel; 4 Department of Psychology, Emory University, Atlanta, GA 30322 USA; 5 Department of Psychiatry and Behavioral Sciences, Emory University, Atlanta, GA 30322 USA

**Keywords:** Diagnostic markers, Human behaviour

## Abstract

Investigations of pathophysiological mechanisms implicated in vulnerability to depression have been negatively impacted by the significant heterogeneity characteristic of psychiatric syndromes. Such challenges are also reflected in numerous null findings emerging from genome-wide association studies (GWAS) of depression. Bolstered by increasing sample sizes, recent GWAS studies have identified genetics variants linked to MDD. Among them, Okbay and colleagues (Nat. Genet. 2016 Jun;48(6):624–33) identified genetic variants associated with three well-validated depression-related phenotypes: subjective well-being, depressive symptoms, and neuroticism. Despite this progress, little is known about psychopathological and neurobiological mechanisms underlying such risk. To fill this gap, a genetic risk score (GRS) was computed from the Okbay’s study for a sample of 88 psychiatrically healthy females. Across two sessions, participants underwent two well-validated psychosocial stressors, and performed two separate tasks probing reward learning both before and after stress. Analyses tested whether GRS scores predicted anhedonia-related phenotypes across three units of analyses: self-report (Snaith Hamilton Pleasure Scale), behavior (stress-induced changes in reward learning), and circuits (stress-induced changes in striatal reward prediction error; striatal volume). GRS scores were negatively associated with anhedonia-related phenotypes across all units of analyses but only circuit-level variables were significant. In addition, the amount of explained variance was systematically larger as variables were putatively closer to the effects of genes (self-report < behavior < neural circuitry). Collectively, findings implicate anhedonia-related phenotypes and neurobiological mechanisms in increased depression vulnerability, and highlight the value of focusing on fundamental dimensions of functioning across different units of analyses.

## Introduction

Progress in elucidating the pathophysiology and etiology of major depressive disorder (MDD) has been hampered by the substantial heterogeneity of this prevalent disorder. In an attempt to overcome these challenges, in 2010, the US National Institute of Mental Health launched the Research Domain Criteria (RDoC) initiative^1,2^, in which fundamental dimensions of behaviors are parsed into domains (e.g., positive valence systems) and subdomains (e.g., reward learning) that map onto precise behavioral and neurobiological variables. Within this conceptual framework, domains and subdomains are investigated across units of analyses—genes → molecules → cells → circuits → physiology → behavior → self-report—and are hypothesized to be critically implicated across traditional diagnostic syndromes.

Anhedonia—defined as the loss of pleasure or interest in previously rewarding stimuli—has emerged as an important phenotype critically implicated in various disorders, including major depression, schizophrenia, substance use disorders, and post-traumatic stress disorders^3,4^, among others. In depression, anhedonia has been found to predict depression^5^ and suicide^6^, and linked to poor response to both pharmacological^7^ and psychological^8^ treatments. Concurrently, convergence across clinical and preclinical data has implicated dopaminergic-rich mesocorticolimbic pathways in anhedonic behaviors^9–11^.

In our efforts to parse the heterogeneity of MDD and develop objective ways to assess anhedonia, we have implemented a laboratory-based assessment of anhedonic behavior probabilistic reward task (PRT)^12^. Rooted in signal-detection theory, the PRT allows to assess reward learning, that is, participants’ ability to modulate behavior as a function of rewards. Of note, we and others have found that reward learning in the PRT was correlated with current and predicted future anhedonic symptoms^12,13^, was blunted in individuals with past or current depression as well as in unaffected offspring of parents with depression^13–16^, and was associated with functional and molecular mesocorticolimbic markers^17–19^.

Directly relevant to the current study, both preclinical and clinical studies have highlighted the role of stress in the emergence of anhedonic behaviors. In preclinical studies, prolonged stressors have been found to induce anhedonic behaviors (e.g., reduced consumption of palatable food, reduced reward learning in a rodent version of the PRT^11,20^) and abnormalities within the mesocorticolimbic pathways, including profound dopaminergic downregulation within the nucleus accumbens (NAc) and striatal regions^9,10^. In humans, exposure to both acute and chronic stressors induced increased self-reported anhedonic symptoms^21^, reduced reward learning as assessed by the PRT^22–24^, and disrupted reward prediction errors (RPEs) in the NAc^25^. Moreover, both among healthy and depressed samples, self-reported anhedonia was inversely related to bilateral NAc and putamen volume^26–28^.

Of note, stress-induced reduction in reward learning or striatal RPE in healthy controls mirrored similar effects emerging when comparing unmedicated individuals with MDD to healthy controls under baseline (no-stress) conditions, indicating that stress-induced anhedonia might be a promising phenotype linked to increased vulnerability to depression (for review, see ref. ^29^). A focus on objectively assessed phenotypes might be particularly useful in genetics studies of MDD, which—with the exception of recent reports^30,31^—have been characterized by null findings at the genome-wide association level^32,33^. Recently, Okbay et al.^34^ performed a genome-wide association study (GWAS) by aggregating data across subjective well-being, depressive symptoms, and neuroticism (a well-known risk factor for MDD). Using a large sample (*N* = 298,420), they found that these three phenotypes were strongly genetically correlated (*ρ* ≈ 0.8); critically, they also identified several variants associated with these depression-relevant phenotypes. Although valuable, these associations do not pinpoint mechanisms that might confer increased depression vulnerability.

In the current study, we investigated whether a genetic risk score (GRS) computed using the variants emerging from the Okbay’s study predicted anhedonia-related markers across three units of analysis: self-report, behavior, and brain circuits. To this end, we performed genetics association analyses using data from a recent study in psychiatrically healthy women exposed to laboratory stressors in both a behavioral and imaging session. Prior publications from this sample have focused on links between (1) inflammation and ventral striatal RPE signaling during a reinforcement learning task^25^; and (2) cortisol responses and hippocampal volume^35^. Here, we tested the hypothesis that increased GRS would be associated with markers related to anhedonia across units of analyses. We also evaluated whether the GRS would be differentially linked to a construct depending on the units of analysis under consideration. Specifically, we tested whether the amount of variance explained by the GRS would become increasingly larger as units of analyses become closer to the putative effects of genes (i.e., variance explained: self-report < behavior < brain circuitry). Note that this assumption is consistent with central tenets of endophenotypic conceptualizations of psychopathology^36,37^ and imaging genetics^38,39^ and the hope that, by focusing on intermediate psychopathological and biological (endo)phenotypes, we may “move closer to the DNA level”^40^ (p. 789). Although intuitive, these tenets have been challenging to confirm^40–43^.

## Methods

### Participants

Eighty-eight psychiatrically, medically and neurologically healthy female participants recruited from the community through various advertisement took part in a behavioral session, in which they performed the PRT both before and after a psychosocial stressor (see below)^35^. Before any procedures were administered, participants provided written informed consent to a protocol approved by the Partners HealthCare Human Research Committee. The sample size was based on power analyses based on effect sizes from prior studies probing stress-induced reduction in reward learning^22,23^, highlighting that a sample exceeding *N* = 80 would provide > 0.85 power to detect effects. All participants were right-handed, nonsmokers; their mean age was 26 (SD = 5.3), and their mean years of education was 16.5 (SD = 1.7). Totally, 69% were Caucasian, 92% non-Hispanic (see also Supplementary Table 1). Of the 88 participants, 75 completed a neuroimaging session, in which they performed an instrumental reinforcement learning task before and after a different psychosocial stressor^25^. To avoid sex-dependent differences in stress reactivity^44^, only females were included. Participants were excluded if they reported current or past psychiatric disorder (as assessed by a Structured Clinical Interview for the DSM-IV, SCID^45^), >five lifetime exposures to any illegal substance (potential recent use was evaluated using a urine drug test), psychotropic medications or being a current smoker. Of the 88 participants, 83 were available for DNA analyses.

### Study procedures

The study involved two laboratory visits, both taking place between 11 a.m. and 4 p.m. to minimize diurnal cortisol variations^46^. During the first visit, a masters-level clinician performed the SCID to confirm eligibility. Participants then provided plasma and saliva samples for DNA and cortisol analyses, respectively, and completed the Snaith–Hamilton Pleasure Scale (SHPS^47^) to assess anhedonia, among other scales. Next, participants completed two tasks, including the PRT, before stress (prestress administration). Subsequently, they underwent a psychosocial stressor (Maastricht acute stress test (MAST)). Immediately thereafter, participants repeated the PRT (poststress administration).

After the first session, participants returned within a month (on average, 25 ± 21 days) to complete the functional magnetic resonance imaging (fMRI) session. This session included a second stress paradigm (Montreal imaging stress task (MIST)) interleaved with six blocks of an instrumental reinforcement learning task, with two runs for each stress condition (runs 1 and 2: prestress; runs 3 and 4: during-stress; runs 5 and 6: poststress), thus enabling us to assess the impact of stress on RPE modeling^25^.

### Genotyping and computation of GRS

DNA was extracted from venous whole blood. Genotyping was performed at the Stanley Center for Psychiatric Research at the Broad Institute using the Illumina Infinium PsychArray BeadChip and Birdsee calling algorithm^48^. Single nucleotide variants (SNVs) were excluded when missing genotypes per SNVs > 5% and missing SNV genotypes per individual > 2%. GRS analyses were performed in PLINK^49^ based on established procedures described elsewhere^50^ using imputed genotype data. Imputation was performed using the 1000 Genomes Project Data with an imputation quality score of INFO > 0.8 retained for analysis. The GRS was generated based on the summary statistics from the top 22 SNVs derived from the GWAS of subjective well-being (*n* = 298,420), depressive symptoms (*n* = 180,866) and neuroticism (*n* = 170,911) by Okbay et al.^34^. After imputation and quality control, the dataset comprised 14 of the 22 SNVs (see Supplementary Table 2). For each SNV we retained the risk allele and the effect size of the association measured as a regression beta value. The weighted GRS was generated using the logarithmic-transformed version of the beta value.

### MAST (behavioral session)

The MAST^51^ included a 10-min acute stress phase that combines the physical aspects of immersing one hand in ice water (1–3 °C) from the cold pressor test, with the unpredictability, uncontrollability, negative social-feedback, and mental arithmetic elements of the trier social stress test. To prolong stress, immediately upon MAST completion participants were told by a nonemphatic male study staff that their performance in the math portion was not good enough and that they would need to repeat the task afterwards. Later, participants were informed that repeating the task was not necessary since their performance was “good enough” (i.e., relief was provided). As described in details in recent publications from the current sample^25,35^, our modified version of the MAST produced highly significant increase in salivary cortisol, negative affect, and state anxiety as well as decreases in positive affect.

### MIST (imaging session)

During fMRI, stress was induced using a modified version of the MIST^52^. This task requires participants to complete arithmetic problems of varying difficulty while their performance was publicly evaluated by strangers. Problems vary in terms of time allotted and difficulty level such that “Easy” runs involved only simple arithmetic problems (e.g., 4 − 0 + 2), whereas “Hard” runs involved more difficult problems and shorter response times (e.g., 65/15 + 27/3). Participants were instructed that they had to maintain an 80% accuracy level. In reality, maintaining 80% was very easy for Easy blocks and made impossible for Hard blocks by increasing difficulty and reducing response times according to individual participants’ accuracy. After Hard blocks, participants were presented with a prerecorded video that was made to believe as a live video-conference call, which showed an unfriendly and impatient experimenter who complained that their performance was not adequately maintained at the 80% level. As described in details in a recent publication from the current sample^25^, our modified version of the MIST produced highly significant increase in negative affect and decreases in positive affect.

### PRT (behavioral session)

The PRT has been described in detail elsewhere^12,13,53^. In each trial, participants were asked to indicate whether a short or long mouth (or nose) was presented by pressing one of two keys (counterbalanced across subjects). The task included 160 trials, divided into two 80-trial blocks. Each trial started with the presentation of a fixation cross (750–900 ms), followed by a mouthless (or noseless) cartoon face (500 ms); next, either a short or long mouth (or nose) was presented for 100 ms, and the mouthless (or noseless) cartoon face remained for 500 ms. In each block, 32 correct responses were followed by positive feedback (“Correct!! You won 20 cents”), which was displayed for 1500 ms in the center of the screen, followed by a blank screen for 250 ms. To induce a response bias, an asymmetrical reinforcer ratio was used, such that correct responses for the rich stimulus were rewarded three times more frequently than correct responses for the other (“lean”) stimulus (i.e., 24 vs. 8 rewards, respectively). Participants were informed that not all correct responses would be rewarded but were not told that one of the stimuli would be rewarded more frequently.

### Instrumental learning task (imaging session)

Interleaved within the “Easy” and “Hard” blocks of the MIST were functional MRI runs of a separate instrumental conditioning task^54^. This well-validated paradigm was used to measure RPE signals in the ventral striatum. For each trial, participants were instructed to choose between two visual stimuli. Each of the stimulus pairs was associated with a given outcome (gain: win $1 or $0; loss: lose $1 or $0; neutral: look at gray square or nothing). For gain and loss pairs, the probabilities of winning $1/$0 varied between 80/20% and 20/80% for each stimulus in the pair. In the neutral pair, there was no monetary outcome. For each trial, one pair was randomly presented, with one stimulus above and one below a fixation cross (counterbalanced). The subject was instructed to choose the upper or lower stimulus by pressing one of two keys. After a jittered delay interval, participants received feedback (either “Nothing”, “Gain”, “Loss” or a gray square with no monetary value for neutral trials). Each run lasted approximately 4 min and consisted of 36 trials (12 per condition). There was a total of six RL runs, with two runs for each stress condition (“Pre-Stress”, “During-Stress”, and “Post-Stress”).

### Computational RL model

To estimate prediction errors, a standard Q-learning model was fit to participants’ choice data. For this model, individual choices and outcomes for each pair of stimuli, A and B, were entered into a Q-learning algorithm to estimate the expected values of choosing stimulus A (Qa) or stimulus B (Qb)^55^. For each condition (e.g., prestress and poststress), *Q* values were initialized at 0. For every subsequent trial *t*, the value of the chosen stimulus (A or B) was updated according to the rule *Qa*(*t* + 1) = *Qa*(*t*) + α*δ(*t*), where *δ*(*t*) represented a prediction error [*δ*(*t*) = *R*(*t*) − *Qa*(*t*)], which was operationalized as the difference between the expected outcome [*Q*(*t*)] and the actual outcome [*R*(*t*)]. The reinforcement magnitude *R* was set to be +1, −1 and 0 for winning, losing, and neutral outcomes, respectively. Based on the *Q* value for each option at each trial, the probability of selecting an option was then estimated using the softmax selection rule^56^

$${Pa}\left( {t} \right) = {\mathrm{exp}}\left( {{Qa}\left( {t} \right){\mathrm{/beta}}} \right){\mathrm{/}}\left( {{\mathrm{exp}}\left( {{Qa}\left( {t} \right){\mathrm{/beta}}} \right)} \right) + {\mathrm{exp}}\left( {{Qb}\left( {t} \right){\mathrm{/beta}}} \right).$$

For generation of prediction error-signals for analyses, predetermined alpha and beta parameters were drawn from a prior study using this paradigm^54^, in keeping with the recommendation to use population-level free-parameters for the purpose of fMRI modeling^57^. Consistent with best-fitting learning rate (alpha) parameters identified by Pessiglione and colleagues^54^, we observed a best-fitting group-averaged alpha of 0.28 (vs. 0.29 as reported in ref. ^54^) for gain pairs and 0.46 for loss pairs (identical to that reported in ref. ^54^). For temperature (beta) parameters, we observed a group-averaged optimal beta of 2.24 for gain pairs and 5.23 for loss pairs.

### fMRI acquisition

Data were acquired using a 3-T Siemens Tim Trio scanner with a 32-channel head coil at the McLean Imaging Center. Trial presentation was synchronized to initial volume acquisition. Scanning protocol included low- and high-resolution structural images using standard parameters. Functional (T2* weighted) images were acquired using a GRAPPA EPI protocol with the following parameters: TR = 3000 ms, TE = 30 ms, flip angle = 75°, FOV = 224 × 224 × 170 mm with 57 interleaved axial slices. High-resolution T1-weighted MPRAGE images [TR = 2200 ms; TE = 1.54 ms; FOV = 230 mm; matrix = 192 × 192; resolution = 1.22 mm^3^; 144 slices] were collected for volumetric analyses.

### Neuroimaging analysis

Neuroimaging data were analyzed using SPM8 (http://www.fil.ion.ucl.ac.uk/spm/software/spm8/). For functional analyses, preprocessing in SPM8, included slice timing correction, realignment estimation, and implementation, normalization to MNI space, and spatial smoothing using a 6 mm Gaussian kernel. For single-subject fixed-effects models, a single GLM was used to estimate BOLD signal across the 6 runs that separately modeled the cue and feedback phases for win, loss, and neutral trials. To examine neural RPE signals, model-derived estimates of trial-wise prediction errors were entered as a parametric modulator (pmod) during trial feedback for win and loss trials. This pmod contrast representing RPE signals across all runs was then entered into a random-effects analysis to examine the main effect of PE signals across all stress conditions. To examine the effects of stress on RPE signaling, additional random-effects models were tested examining the interactions between RPE prestress vs. poststress runs. Beta weights were extracted from a priori ROIs for further analyses on effects of stress on RPE signaling. A positive-RPE beta indicates higher activation for unexpected reward and lower activation for unexpected omission of rewards during gain trials.

### Regions-of-interest (ROI) analyses

**Fig. 1:**
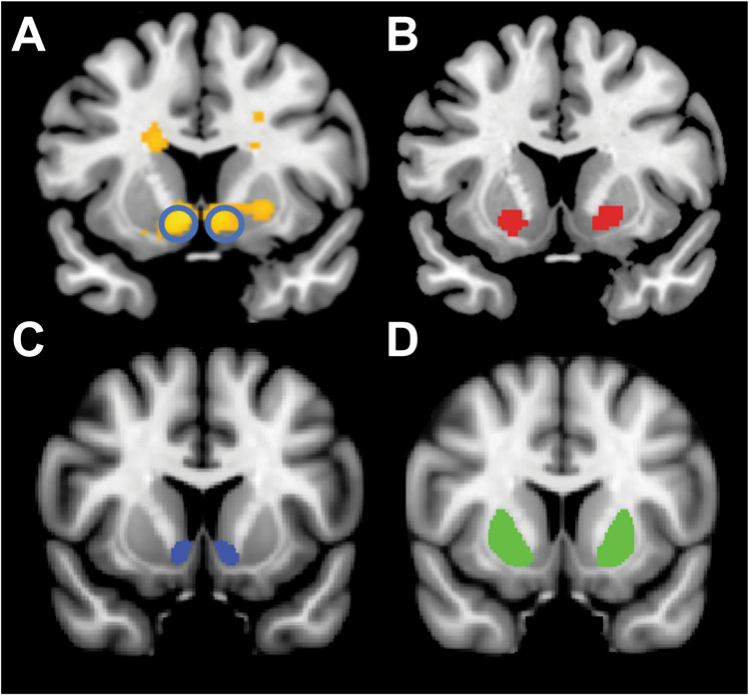
Coronal slices showing the functional and structural regions-of-interest (ROIs) considered for the analyses. **a** Functional (bilateral) nucleus accumbens ROI (yellow color) previously found to be associated with stress-induced RPE reductions^25^. **b** Functional (bilateral) putamen ROI (red color) emerging from a meta-analysis of RPE studies in healthy controls^60^. **c** Structural (bilateral) nucleus accumbens (yellow color) and putamen (red color) ROI. For analyses, structural ROIs were extracted for each participant individually and entered into analyses. For panel **a**, circles highlight the nucleus accumbens ROI that was considered for analyses

Based on prior findings, two ROIs were tested. The first was the bilateral NAc region emerging from prior analyses of this sample to be associated with stress-induced RPE reductions^25^ (Fig. 1a). (One participant was an outlier in stress-induced NAc activation and thus was omitted from the analyses.) The second included the bilateral putamen due to evidence of (1) reduced RPE signals in the putamen in MDD^58^; (2) correlations between reduced RPE in the putamen and depressive symptoms^59^; and (3) links between smaller bilateral putamen and anhedonia among adolescents^28^. Anatomically constrained bilateral putamen were extracted from a recent meta-analysis of RPE studies in healthy controls^60^ (Fig. 1b). A Bonferroni correction (*p* = 0.05/2 = 0.025) was used.

### Structural analyses

Volumetric segmentation was performed using FreeSurfer^61^ (v5.3). The brain parcellation and segmentation were run using the standard “recon-all” script using default settings. For each subject, the postprocessing output was thoroughly inspected for segmentation errors and no manual edits were required. Intracranial volume was calculated to correct for interindividual differences in total brain size. To parallel the functional analyses, bilateral volume for the NAc (Fig. 1c) and putamen (Fig. 1d) were extracted.

### Statistical analysis

Genetic analyses were performed in the Linux environment. Imputation was performed using the molgenis-imputation software, which provides rapid generation of genetic imputation scripts for grid/cluster/local environments using SHAPEIT for phasing, Genotype Harmonizer for quality control and impute2 tool for imputation (https://github.com/molgenis/molgenis-imputation)^62^. GRS was generated using PLINK and the association analysis with phenotypic variables of interest were performed in R. Plots were generated using the R-package “ggplot2” (http://ggplot2.tidyverse.org/reference/).

### GRS analyses

We used GWAS summary results from Okbay et al.^34^ to derive GRS (see Supplementary Table 2). Next, six hierarchical regression analyses were performed to evaluate whether GRS predicted anhedonia-related measures assessed across units of analysis: self-report (SHAPS score), behavior (stress-induced reduction in reward learning), and brain circuits. Brain circuit was probed both at the functional (stress-induced reduction in striatal RPE) and structural (striatal volume) level. For all analyses, ancestry-related variables (i.e., PC1 and PC2 scores) were entered in the first step, followed by GRS score in the second step. Unique variance explained by the GRS after accounting for ancestry-related variables is reported. Statistical analyses were performed using SPSS (version 24.0).

## Results

**Fig. 2:**
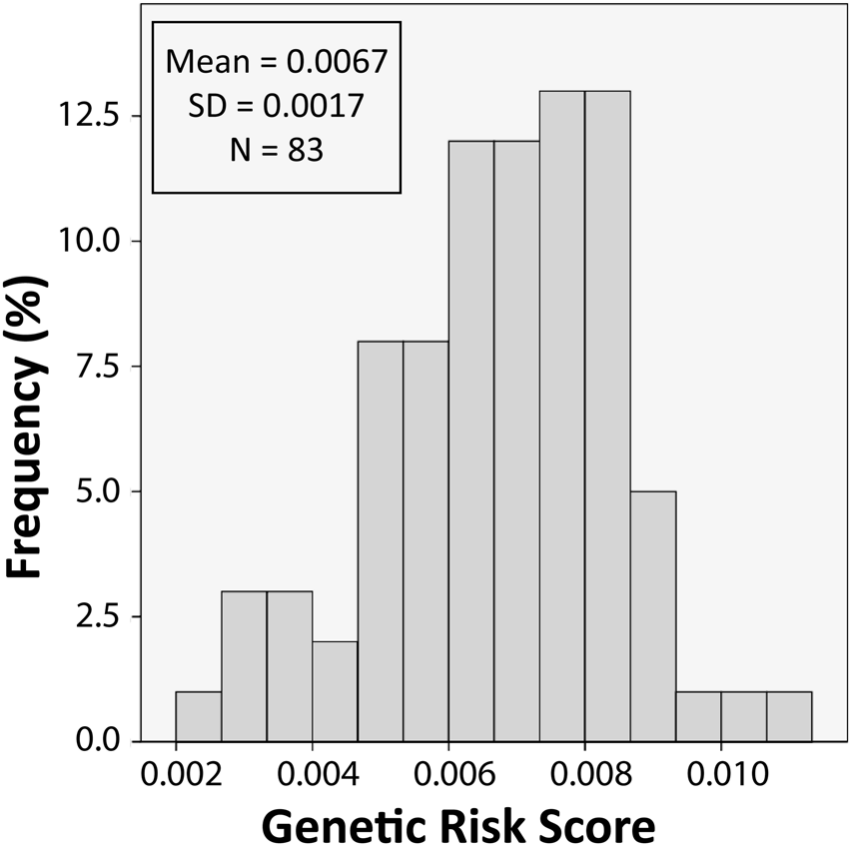
Histogram of genetic risk scores across 83 female participants

GRS was available for 83 participants; the distribution of GRS was normally distributed (Fig. 2). Table 1 summarizes the findings emerging from the hierarchical regression analyses, whereas Supplementary Table 3 summarizes intercorrelations among units of analysis.

**Table 1:** Summary of statistical associations between genetic risk score and anhedonia-related markers across units of analyses (self-report, behavior, and brain circuit)

Units of analyses	Measure	*N*	Δ*R*^2^	Δ*F*, *p* value
Self-report	Snaith Hamilton pleasure scale score	82	0.017	1.39, 0.24
Behavior	Stress-induced changes in reward learning	59	0.035	2.31, 0.135
Circuits (functional)	Stress-induced changes in RPE Bilateral NAc RPE (poststress–prestress) Bilateral Put RPE (poststress–prestress)	62^a^ 63	0.065 0.074	4.76, 0.033 5.14, 0.027
Circuits (structural)	Striatal volume Bilateral NAc volume Bilateral Put volume	73 73	0.064 0.095	5.01, 0.028 7.32, 0.009

*RPE* reward prediction error, *NAc* nucleus accumbens, *Put* putamen

^a^One participant was an outlier in stress-induced NAc activation and was omitted from the analyses

*Self-report measure of anhedonia (SHAPS score)*: The GRS did not significantly predict SHAPS scores (Δ*R*^2^ = 0.017, *p* > 0.24).

*Behavioral measure of anhedonia (stress-induced changes in reward learning)*: The GRS did not significantly predict stress-induced changes in reward learning ([response bias (Block 2) − response bias (Block 1)]_poststress_ − [response bias (Block 2) − response bias (Block 1)]_prestress_) (Δ*R*^2^ = 0.035, *p* > 0.12). For the main effect of stress on response bias, see “Supplement”.

**Fig. 3:**
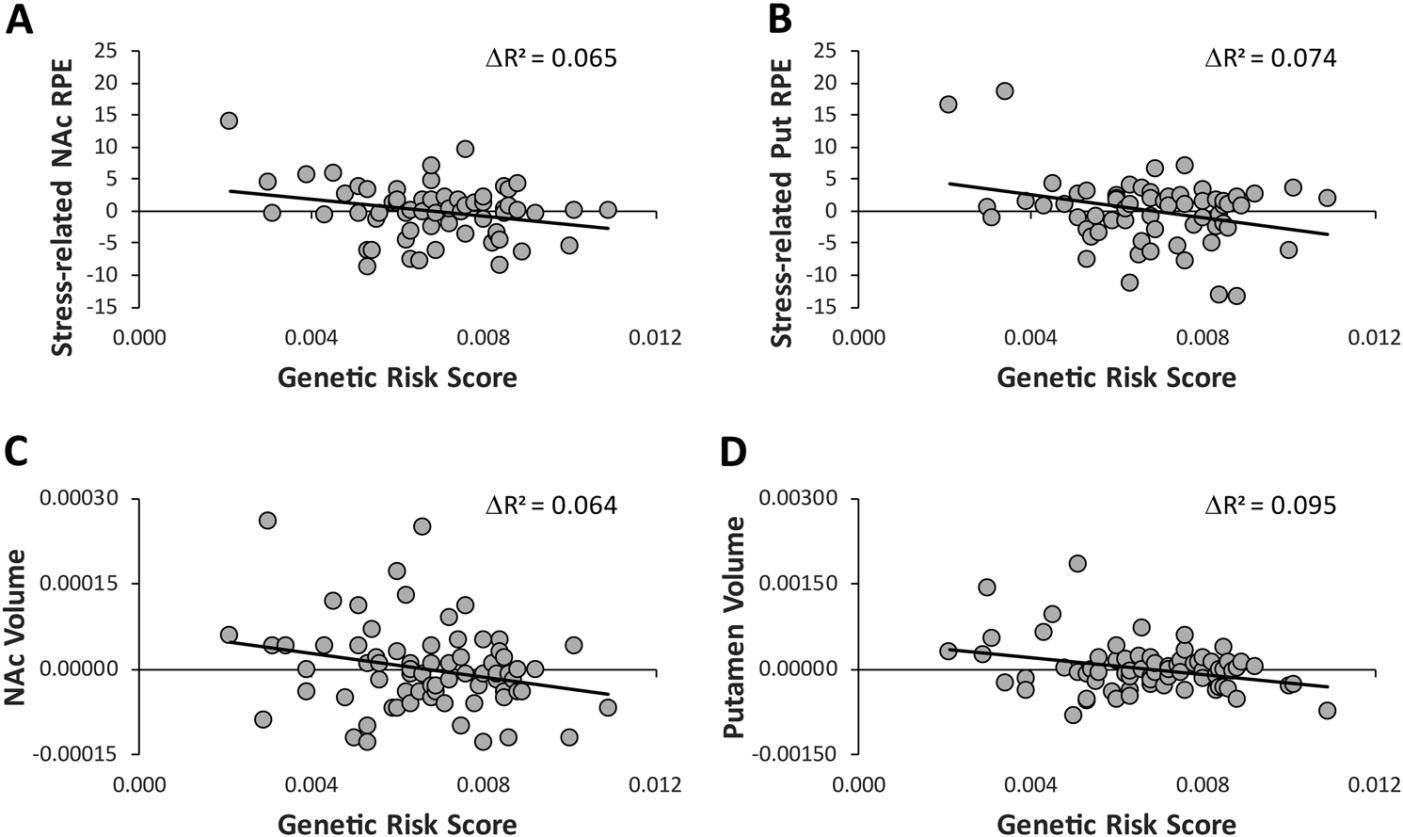
Scatterplots of associations between depression-related genetic risk scores and **a** bilateral nucleus accumbens stress-induced RPE changes (Δ*R*^2^ = 0.065, *p* = 0.033); **b** bilateral putamen stress-induced RPE changes (Δ*R*^2^ = 0.074, *p* = 0.027); **c** bilateral nucleus accumbens volume (Δ*R*^2^ = 0.095, *p* = 0.028); and **d** bilateral putamen volume (Δ*R*^2^ = 0.095, *p* = 0.009). For the anhedonia markers, residualized values are plotted (removing variance associated with ancestry-related variables (i.e., PC1 and PC2 scores)). For the volumetric data, intracranial volume was calculated to correct for interindividual differences in total brain size

*Neural (functional) measures of anhedonia (stress-induced changes in striatal RPE)*: The regression analyses indicated that GRS predicted both bilateral NAc (Δ*R*^2^ = 0.065, Δ*F*(1,58) = 4.73, *p* = 0.033) and bilateral putamen (Δ*R*^2^ = 0.074, Δ*F*(1,58) = 5.14, *p* = 0.027) stress-induced RPE changes ([RPE]_poststress_ − [RPE]_prestress_). Individuals with a higher genetic risk exhibited higher stress-induced reduction in RPE in the bilateral NAc and putamen (Fig. 3a, b).

*Neural (structural) measures of anhedonia (striatal volume)*: The regression analyses indicated that GRS predicted both bilateral NAc (Δ*R*^2^ = 0.095, Δ*F*(1,69) = 5.01, *p* = 0.028) and bilateral putamen (Δ*R*^2^ = 0.095, Δ*F*(1,69) = 7.32, *p* = 0.009) volume. Individuals with a higher genetic risk exhibited smaller bilateral NAc and putamen volume (Fig. 3c, d).

**Fig. 4:**
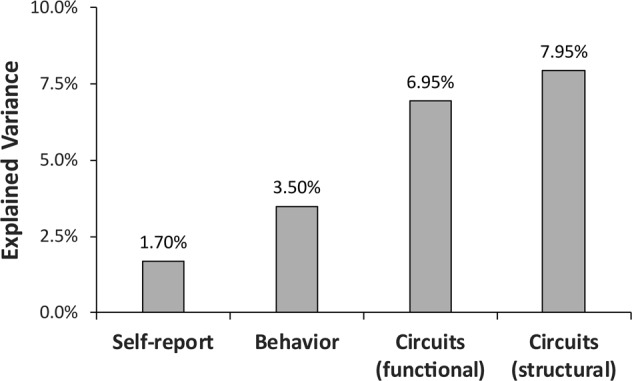
Amount of variance explained by the genetic risk score for anhedonia-related markers across units of analysis (self-report → behavior → brain circuits)

A comparison across units of analyses indicated that the amount of variance GRS explained increased gradually from self-report → behavior → circuits (Fig. 4). Consistent with this observation, a final set of hierarchical regression analyses entering ancestry-related variables in the first step, SHAPS scores and stress-induced changes in reward learning in the second step, and GRS in the third step indicated that GRS explained significant unique variance for all four circuits-level variables [NAc RPE: Δ*R*^2^ = 0.170, Δ*F*(1,49) = 11.19, *p* = 0.002; Putamen RPE: Δ*R*^2^ = 0.081, Δ*F*(1,49) = 4.64, *p* = 0.036; NAc volume: (Δ*R*^2^ = 0.076), Δ*F*(1,57) = 4.88, *p* = 0.031; Putamen volume: Δ*R*^2^ = 0.122, Δ*F*(1,57) = 8.09, *p* = 0.006].

## Discussion

GWAS have proven successful in identifying common single-nucleotide polymorphisms (SNPs) associated with disease risks for psychiatric disorders, including schizophrenia^63^, bipolar disorder^64^, autism spectrum disorder^65^, and very recently, MDD^30,66^. GWAS have also demonstrated that a substantial proportion of the heritability of MDD is explained by a polygenic component consisting of thousands of common SNPs of small effect and overlapping polygenic risk among schizophrenia, bipolar, and MDD disorders, indicating pleiotropic effects of some risk variants across traditional diagnostic classifications^67,68^. Recently, large-scale GWAS analyses have identified a number of genetic variants robustly associated with depressive symptoms and subjective well-being^34,69^. Results of these studies also confirm the highly polygenetic and heterogenous nature of depressive phenotype. Although these results are statistically robust, the functional effects of these variants remain unclear. Mapping the effects of risk genes on distinct domains of brain function and structure can provide important biological insights into the mechanisms by which these genes may confer illness risk^70^.

Here, we tested the novel hypothesis that, among young, psychiatrically healthy women, GRS linked to depression-related phenotypes^34^ would predict anhedonic phenotypes across three units of analysis: self-report, behavior, and brain circuits. This hypothesis was derived from a convergence of clinical evidence emphasizing that anhedonia plays a critical role in vulnerability to, disease course of, and treatment for MDD^5,7,8,29^ and preclinical literature emphasizing the role of stress-induced disruptions in mesolimbic pathways in the emergence of anhedonic phenotypes and depression vulnerability^9,10,20,29^. Two main findings emerged. First, although GRS was negatively associated with anhedonia-related markers across all three units of analyses, only brain circuit markers were significantly associated with GRS. Specifically, individuals with a higher genetic risk exhibited higher stress-induced reduction in RPE in the bilateral NAc and putamen as well as smaller bilateral NAc and putamen volume. Second, the amount of explained variance was systematically larger as a function of the hypothesized proximity to the effects of genes: self-report (1.70%) < behavior (3.50%) < circuits (functional) (6.95%), circuits (structural) (7.95%). Notably, stress-induced reduction in RPE in the right NAc and left putamen volume showed the largest explained variance (12.6% and 10.2%, respectively) (data not shown). Highlighting incremental validity, GRS scores continued to predict both functional and structural anhedonia-related markers when accounting for ancestry-related variables as well as both self-reported and behavioral markers of anhedonia.

Mounting evidence implicates dorsal and ventral striatal regions in the pathophysiology of and increased vulnerability to depression. First, hemodynamic, structural and molecular imaging studies have reported abnormalities in both the NAc and putamen in MDD as well as unaffected offspring of parents with MDD^27,58,71–74^. Second, structural MRI studies have linked smaller dorsal striatum to anhedonic symptoms among healthy samples^26,28^. Third, abundant preclinical data have shown that experimental models relevant to depression (which typically involve exposure to uncontrollable and prolonged stressors) are characterized by profound downregulation of mesolimbic dopaminergic pathways implicated in incentive motivation and reinforcement learning^10,11,29^. The current findings add to this literature by showing that genetic variants linked to key depressive phenotypes (well-being, depressive symptoms, and neuroticism) at the GWAS level are associated with individual differences in (1) the propensity to reduce reward prediction errors after exposure to a stressor, and (2) volume of two striatal regions (NAc and putamen) critical for reinforcement learning and motivation. We speculate that such liability might increase risk for depression when facing severe life stressors.

The current study has several strengths, including the focus on an important (endo)phenotype of depression (anhedonia) across multiple units of analyses; the use of two independent and well-validated psychosocial stressors (MAST and MIST); objective assessment of anhedonic phenotypes through laboratory-based tasks (PRT and an instrumental reinforcement learning task); the implementation of computational modeling for fMRI analyses; and the focus on a young, psychiatrically healthy and unmedicated sample, which avoids potential confounds (e.g., medication and prior hospitalization). There are, however, important limitations, including a sample size relatively modest for genetics analyses, which possibly contributed to null findings with respect to self-reported and behavioral markers; the sole focus on a female sample; and the lack of an independent replication sample. The latter point is especially important but we were unable to find a similar dataset involving multiple experimental sessions with psychosocial stressors and reward tasks. Nevertheless, the current findings require independent replication. Moreover, interpretations in terms of effect sizes across units of analysis should be cautious since effect sizes are inversely related to measurement errors^40,43^. For example, the reliability of fMRI data^75,76^ is quite variable and task-dependent, with estimates close to only 0.30. Conversely, structural MRI data^77^ have shown superior reliability, including recent test–retest correlations exceeding 0.95 for measures of cortical thickness^77^ (see also ref. ^78^). Thus, it is also possible that psychometrics features—rather than the hypothesized proximity of a given unit of analysis to the effects of genes—might partially explain the systematic differences in effect sizes. Finally, the current analyses were also repeated by considering (1) a GRS derived from an early report by the MDD Working Group of the Psychiatric GWAS Consortium^32^; (2) a single SNP in the Oprl1 gene (opioid receptor-like 1)^79^; and (3) a genetic profile score combining variants across stress-system genes^80^. Thus, with the exception of bilateral putamen volume (*p* = 0.009), the other results would not survive correction for the four genetic scores considered. In spite of these limitations, the current findings provide novel evidence that GWAS-ascertained genetic variants linked to depression are associated with individual differences in functional and structural features of the key regions within the brain reward system (dorsal and ventral striatum). They also provide corroborating evidence that units of analysis hypothesized to the more proximally related to the effects of genes (e.g., brain circuits) are more strongly linked to genetic risks.

## Supplementary information

Related Manuscript File

## Data Availability

Data are available at the NIMH Data Archive (https://nda.nih.gov/edit_collection.html?id=2366).

## Code availability

Analysis scripts are available upon request.

## References

[1] Insel T (2010). Research domain criteria (RDoC): toward a new classification framework for research on mental disorders. Am. J. Psychiatry.

[2] Cuthbert BN (2014). The RDoC framework: facilitating transition from ICD/DSM to dimensional approaches that integrate neuroscience and psychopathology. World Psychiatry.

[3] Whitton AE, Treadway MT, Pizzagalli DA (2015). Reward processing dysfunction in major depression, bipolar disorder and schizophrenia. Curr. Opin. Psychiatry.

[4] Nawijn L (2015). Reward functioning in PTSD: a systematic review exploring the mechanisms underlying anhedonia. Neurosci. Biobehav. Rev..

[5] Wardenaar KJ, Giltay EJ, van Veen T, Zitman FG, Penninx BWJH (2012). Symptom dimensions as predictors of the two-year course of depressive and anxiety disorders. J. Affect Disord..

[6] Fawcett J (1990). Time-related predictors of suicide in major affective disorder. Am. J. Psychiatry.

[7] Spijker J, Bijl RV, de Graaf R, Nolen WA (2001). Determinants of poor 1-year outcome of DSM-III-R major depression in the general population: results of the Netherlands Mental Health Survey and Incidence Study (NEMESIS). Acta Psychiatr. Scand..

[8] McMakin DL (2012). Anhedonia predicts poorer recovery among youth with selective serotonin reuptake inhibitor treatment-resistant depression. J. Am. Acad. Child Adolesc. Psychiatry.

[9] Anisman H, Matheson K (2005). Stress, depression, and anhedonia: caveats concerning animal models. Neurosci. Biobehav Rev..

[10] Cabib S, Puglisi-Allegra S (2012). The mesoaccumbens dopamine in coping with stress. Neurosci. Biobehav. Rev..

[11] Der-Avakian A, Markou A (2012). The neurobiology of anhedonia and other reward-related deficits. Trends Neurosci..

[12] Pizzagalli DA, Jahn AL, O’Shea JP (2005). Toward an objective characterization of an anhedonic phenotype: a signal-detection approach. Biol. Psychiatry.

[13] Pizzagalli DA, Iosifescu D, Hallett LA, Ratner KG, Fava M (2008). Reduced hedonic capacity in major depressive disorder: evidence from a probabilistic reward task. J. Psychiatr. Res..

[14] Pechtel P, Dutra SJ, Goetz EL, Pizzagalli DA (2013). Blunted reward responsiveness in remitted depression. J. Psychiatr. Res..

[15] Whitton AE (2016). Blunted neural responses to reward in remitted major depression: a high-density event-related potential study. Biol. Psychiatry Cogn. Neurosci. Neuroimaging..

[16] Liu W (2016). Anhedonia is associated with blunted reward sensitivity in first-degree relatives of patients with major depression. J. Affect. Disord..

[17] Kaiser RH (2018). Frontostriatal and dopamine markers of individual differences in reinforcement learning: a multi-modal investigation. Cereb. Cortex..

[18] Santesso DL (2009). Single dose of a dopamine agonist impairs reinforcement learning in humans: evidence from event-related potentials and computational modeling of striatal-cortical function. Hum. Brain Mapp..

[19] Vrieze E (2013). Measuring extrastriatal dopamine release during a reward learning task. Hum. Brain Mapp..

[20] Der-Avakian A (2017). Social defeat disrupts reward learning and potentiates striatal nociceptin/orphanin FQ mRNA in rats. Psychopharmacology.

[21] Berenbaum H, Connelly J (1993). The effect of stress on hedonic capacity. J. Abnorm. Psychol..

[22] Bogdan R, Pizzagalli DA (2006). Acute stress reduces reward responsiveness: implications for depression. Biol. Psychiatry.

[23] Bogdan R, Santesso DL, Fagerness J, Perlis RH, Pizzagalli DA (2011). Corticotropin-releasing hormone receptor type 1 (CRHR1) genetic variation and stress interact to influence reward learning. J. Neurosci..

[24] Nikolova Y, Bogdan R, Pizzagalli DA (2012). Perception of a naturalistic stressor interacts with 5-HTTLPR/rs25531 genotype and gender to impact reward responsiveness. Neuropsychobiology.

[25] Treadway MT (2017). Association between interleukin-6 and striatal prediction-error signals following acute stress in healthy female participants. Biol. Psychiatry.

[26] Harvey PO, Pruessner J, Czechowska Y, Lepage M (2007). Individual differences in trait anhedonia: a structural and functional magnetic resonance imaging study in non-clinical subjects. Mol. Psychiatry.

[27] Pizzagalli DA (2009). Reduced caudate and nucleus accumbens response to rewards in unmedicated individuals with major depressive disorder. Am. J. Psychiatry.

[28] Auerbach RP (2017). Neuroanatomical prediction of anhedonia in adolescents. Neuropsychopharmacology.

[29] Pizzagalli DA (2014). Depression, stress, and anhedonia: toward a synthesis and integrated model. Annu Rev. Clin. Psychol..

[30] Howard DM (2019). Genome-wide meta-analysis of depression identifies 102 independent variants and highlights the importance of the prefrontal brain regions. Nat. Neurosci..

[31] Cai N (2015). Sparse whole-genome sequencing identifies two loci for major depressive disorder. Nature.

[32] Ripke S, Major Depressive Disorder Working Group of the Psychiatric GWAS Consortium (2013). A mega-analysis of genome-wide association studies for major depressive disorder. Mol. Psychiatry.

[33] Levinson DF (2014). Genetic studies of major depressive disorder: why are there no genome-wide association study findings and what can we do about it?. Biol. Psychiatry.

[34] Okbay A (2016). Genetic variants associated with subjective well-being, depressive symptoms and neuroticism identified through genome-wide analyses. Nat. Genet..

[35] Admon R (2017). Distinct trajectories of cortisol response to prolonged acute stress are linked to affective responses and hippocampal gray matter volume in healthy females. J. Neurosci..

[36] Gottesman II, Gould TD (2003). The endophenotype concept in psychiatry: etymology and strategic intentions. Am. J. Psychiatry.

[37] Hasler G, Drevets WC, Manji HK, Charney DS (2004). Discovering endophenotypes for major depression. Neuropsychopharmacology.

[38] Meyer-Lindenberg A, Weinberger DR (2006). Intermediate phenotypes and genetic mechanisms of psychiatric disorders. Nat. Rev. Neurosci..

[39] Hariri AR, Drabant EM, Weinberger DR (2006). Imaging genetics: perspectives from studies of genetically driven variation in serotonin function and corticolimbic affective processing. Biol. Psychiatry.

[40] Kendler KS, Neale MC (2010). Endophenotype: a conceptual analysis. Mol. Psychiatry.

[41] Bogdan R (2017). Imaging genetics and genomics in psychiatry: a critical review of progress and potential. Biol. Psychiatry.

[42] Franke B (2016). Genetic influences on schizophrenia and subcortical brain volumes: large-scale proof of concept. Nat. Neurosci..

[43] Flint J, Munafo MR (2007). The endophenotype concept in psychiatric genetics. Psychol. Med..

[44] Kudielka BM, Kirschbaum C (2005). Sex differences in HPA axis responses to stress: a review. Biol. Psychol..

[45] First MB, Spitzer RL, Gibbon M, Williams JBW (1994). Structured Clinical Interview for Axis 1 DSM-IV Disorders..

[46] Blascovich J., Vanman E. J., Berry Mendes W., Dickerson S. *Social Psychophysiology for Social and Personality Psychology*. (Sage Publications, 2011). 160 p.

[47] Snaith RP (1995). A scale for the assessment of hedonic tone the Snaith-Hamilton Pleasure Scale. Br. J. Psychiatry.

[48] Korn JM (2008). Integrated genotype calling and association analysis of SNPs, common copy number polymorphisms and rare CNVs. Nat. Genet..

[49] Purcell S (2007). PLINK: a tool set for whole-genome association and population-based linkage analyses. Am. J. Hum. Genet..

[50] Purcell SM (2009). Common polygenic variation contributes to risk of schizophrenia and bipolar disorder. Nature.

[51] Smeets T (2012). Introducing the Maastricht Acute Stress Test (MAST): a quick and non-invasive approach to elicit robust autonomic and glucocorticoid stress responses. Psychoneuroendocrinology.

[52] Dedovic K (2005). The Montreal Imaging Stress Task: using functional imaging to investigate the effects of perceiving and processing psychosocial stress in the human brain. J. Psychiatry Neurosci..

[53] Tripp G, Alsop B (1999). Sensitivity to reward frequency in boys with attention deficit hyperactivity disorder. J. Clin. Child Psychol..

[54] Pessiglione M, Seymour B, Flandin G, Dolan RJ, Frith CD (2006). Dopamine-dependent prediction errors underpin reward-seeking behaviour in humans. Nature.

[55] Sutton R, Barto A (1998). Reinforcement learning: An introduction..

[56] Luce, R. D. *Individual Choice Behavior: A Theoretical Analysis*. (Wiley, New York, NY, USA)

[57] Daw ND (2011). Trial-by-trial data analysis using computational models. decision making, affect, and learning. Atten. Perform. XXIII.

[58] Kumar P (2018). Impaired reward prediction error encoding and striatal-midbrain connectivity in depression. Neuropsychopharmacology.

[59] Bakker, J. M. et al. From laboratory to life: associating brain reward processing with real-life motivated behaviour and symptoms of depression in non-help-seeking young adults. *Psychol. Med*. 1–11 (2018). 10.1017/S0033291718003446 [Epub ahead of print]. 10.1017/S0033291718003446 PMC6541542 30488820

[60] Chase HW, Kumar P, Eickhoff SB, Dombrovski AY (2015). Reinforcement learning models and their neural correlates: An activation likelihood estimation meta-analysis. Cogn. Affect Behav. Neurosci..

[61] Fischl B (2002). Whole brain segmentation: automated labeling of neuroanatomical structures in the human brain. Neuron.

[62] Kanterakis A (2015). Molgenis-impute: imputation pipeline in a box. BMC Res. Notes.

[63] Schizophrenia Working Group of the Psychiatric Genomics Consortium. (2014). Biological insights from 108 schizophrenia-associated genetic loci. Nature.

[64] Psychiatric GWAS Consortium Bipolar Disorder Working Group. (2011). Large-scale genome-wide association analysis of bipolar disorder identifies a new susceptibility locus near ODZ4. Nat. Genet..

[65] Grove J (2019). Identification of common genetic risk variants for autism spectrum disorder. Nat. Genet..

[66] Wray NR (2018). Genome-wide association analyses identify 44 risk variants and refine the genetic architecture of major depression. Nat. Genet..

[67] Cross-Disorder Group of the Psychiatric Genomics Consortium. (2013). Identification of risk loci with shared effects on five major psychiatric disorders: a genome-wide analysis. Lancet.

[68] Lee SH (2013). Genetic relationship between five psychiatric disorders estimated from genome-wide SNPs. Nat. Genet..

[69] Hyde CL (2016). Identification of 15 genetic loci associated with risk of major depression in individuals of European descent. Nat. Genet..

[70] Hall M-H, Smoller JW (2010). A new role for endophenotypes in the GWAS era: functional characterization of risk variants. Harv. Rev. Psychiatry.

[71] Forbes EE (2009). Altered striatal activation predicting real-world positive affect in adolescent major depressive disorder. Am. J. Psychiatry.

[72] Sharp C (2014). Major depression in mothers predicts reduced ventral striatum activation in adolescent female offspring with and without depression. J. Abnorm. Psychol..

[73] Gotlib IH (2010). Neural processing of reward and loss in girls at risk for major depression. Arch. Gen. Psychiatry.

[74] Pizzagalli Diego A., Berretta Sabina, Wooten Dustin, Goer Franziska, Pilobello Kanoelani T., Kumar Poornima, Murray Laura, Beltzer Miranda, Boyer-Boiteau Anne, Alpert Nathanial, El Fakhri Georges, Mechawar Naguib, Vitaliano Gordana, Turecki Gustavo, Normandin Marc (2019). Assessment of Striatal Dopamine Transporter Binding in Individuals With Major Depressive Disorder. JAMA Psychiatry.

[75] Aron AR, Gluck MA, Poldrack RA (2006). Long-term test-retest reliability of functional MRI in a classification learning task. Neuroimage.

[76] Clément F, Belleville S (2009). Test-retest reliability of fMRI verbal episodic memory paradigms in healthy older adults and in persons with mild cognitive impairment. Hum. Brain Mapp..

[77] Wonderlick JS (2009). Reliability of MRI-derived cortical and subcortical morphometric measures: effects of pulse sequence, voxel geometry, and parallel imaging. Neuroimage.

[78] Elliott LT (2018). Genome-wide association studies of brain imaging phenotypes in UK Biobank. Nature.

[79] Andero R (2013). Amygdala-dependent fear is regulated by Oprl1 in mice and humans with PTSD. Sci. Transl. Med..

[80] Pagliaccio D (2014). Stress-system genes and life stress predict cortisol levels and amygdala and hippocampal volumes in children. Neuropsychopharmacology.

