# Supplementary material for: Depression genetic risk score is associated with anhedonia-related markers across units of analysis
Source: Transl Psychiatry. 2019 Sep 19;9:236. doi: 10.1038/s41398-019-0566-7 (PMC6753161; doi:10.1038/s41398-019-0566-7)
Supplement: Supplementary file 1 — Related Manuscript File [file 41398_2019_566_MOESM1_ESM.docx]

Supplementary Information

**Depression Genetic Risk Score is Associated with Anhedonia-Related Markers Across Units of Analysis**

Guia Guffanti, PhD^1,2^, Poornima Kumar, PhD^1,2^, Roee Admon, PhD^3^, Michael T. Treadway, PhD^4.5^, Mei Hall, PhD^1,2^, Malavika Mehta, BA^2^, Samuel Douglas, BA^2^,

Amanda R. Arulpragasam, MA^4^, Diego A. Pizzagalli, PhD^1,2^

^1^ Department of Psychiatry, Harvard Medical School, Boston, MA 02115

^2^ McLean Hospital, Belmont, MA 02478

^3^ Department of Psychology, University of Haifa, Haifa, Israel

^4^ Department of Psychology, Emory University, Atlanta, GA 30322

^5^ Department of Psychiatry and Behavioral Sciences, Emory University, Atlanta, GA 30322

**Supplementary Methods and Results**

Probabilistic Reward Task (Behavioral Session)

In the main analyses, we focused on stress-induced reduction in reward learning due to abundant preclinical and clinical studies highlighting the role of stress in the emergence of anhedonic behaviors and disruption in mesocorticolimbic pathways critically implicated hedonic behaviors.^1–9^). Such analyses relied on a metric capturing stress-induced changes in reward learning ([Response Bias (Block 2) – Response Bias (Block 1)]_Post-Stress_ - [Response Bias (Block 2) - Response Bias (Block 1)]_Pre-Stress_). Control analyses were performed to test the main effects of stress on response bias. Specifically, a *Stress* (pre-stress, post-stress) x *Block* (Block 1, Block 2) analysis of variance (ANOVA) was performed on response bias scores (N = 59). Contrary to prior findings^6–8^, no effects of Stress emerged (all *F*s < 1.30, all *p*s > 0.25; see Supplementary Fig. 1).

Inter-correlations among units of analysis

Pearson correlation analyses revealed few significant correlations among units of analysis (*Supplementary Table 3*). Poor and even no correlations among self-report, behavioral and physiological variables hypothesized to assess the same construct have been reported in many studies (e.g., ^10–16^), and represent an important conceptual conundrum for studies probing constructs across units of analysis.

**References**

1. Der-Avakian A et al. Social defeat disrupts reward learning and potentiates striatal nociceptin/orphanin FQ mRNA in rats. Psychopharmacology (Berl). 2017;234:1603–14.

2. Der-Avakian A, Markou A. The neurobiology of anhedonia and other reward-related deficits. Trends Neurosci. 2012;35:68–77.

3. Anisman H, Matheson K. Stress, depression, and anhedonia: caveats concerning animal models. Neurosci Biobehav Rev. 2005;29:525–46.

4. Cabib S, Puglisi-Allegra S. The mesoaccumbens dopamine in coping with stress. Neurosci Biobehav Rev. 2012;36:79–89.

5. Berenbaum H, Connelly J. The effect of stress on hedonic capacity. J Abnorm Psychol. 1993;102:474–81.

6. Bogdan R, Pizzagalli DA. Acute stress reduces reward responsiveness: implications for depression. Biol Psychiatry. 2006;60:1147–54.

7. Bogdan R, Santesso DL, Fagerness J, Perlis RH, Pizzagalli DA. Corticotropin-releasing hormone receptor type 1 (CRHR1) genetic variation and stress interact to influence reward learning. J Neurosci. 2011;31:13246–54.

8. Nikolova Y, Bogdan R, Pizzagalli DA. Perception of a naturalistic stressor interacts with 5-HTTLPR/rs25531 genotype and gender to impact reward responsiveness. Neuropsychobiology. 2012;65:45–54.

9. Treadway MT et al. Association between interleukin-6 and striatal prediction-error signals following acute stress in healthy female participants. Biol Psychiatry. 2017;82:570–7.

10. Lang PJ, Levin DN, Miller GA, Kozak MJ. Fear behavior, fear imagery, and the psychophysiology of emotion: the problem of affective response integration. J Abnorm Psychol. 1983;92:276–306.

11. Bradley MM, Lang PJ. Measuring emotion: Behavior, feeling, and physiology. In: Lane RD, Nadel L, editors. Cognitive neuroscience of emotion. New York: Oxford University Press; 2000. p. 242–276.

12. Mauss IB, Levenson RW, McCarter L, Wilhelm FH, Gross JJ. The tie that binds? Coherence among emotion experience, behavior, and physiology. Emotion. 2005;5:175–90.

13. Edelmann RJ, Baker SR. Self-reported and actual physiological responses in social phobia. Br J Clin Psychol. 2002;41:1–14.

14. Lacey JI, Lacey BC. Verification and extension of the principle of autonomic response-stereotypy. Am J Psychol. 1958;71:50–73.

15. Shackman AJ et al. Neural mechanisms underlying heterogeneity in the presentation of anxious temperament. Proc Natl Acad Sci U S A. 2013;110:6145–50.

16. Fernández-Dols J-M, Sánchez F, Carrera P, Ruiz-Belda M-A. Are spontaneous expressions and emotions linked? an Experimental test of coherence. J Nonverbal Behav. 1997;21:163–77.

17. Okbay A et al. Genetic variants associated with subjective well-being, depressive symptoms and neuroticism identified through genome-wide analyses. Nat Genet. 2016;48:624–33.

**Supplementary Figure 1**: Response bias scores as a function of Block and stress manipulation (N = 59).

**Supplementary Table 1.** Sample demographic information

|  | ***n* (%)** |
| --- | --- |
| **Race** |  |
| Caucasian | 61 (69%) |
| Black | 15 (17%) |
| Asian | 10 (12%) |
| Unknown | 2 (2%) |
| **Ethnicity** |  |
| Non-Hispanic | 81 (92%) |
| Hispanic | 5 (6%) |
| Unknown | 2 (2%) |
| **Income** |  |
| <$10,000 | 11 (13%) |
| $10,000–$25,000 | 10 (11%) |
| $25,000–$50,000 | 21 (24%) |
| $50,000–$75,000 | 18 (20%) |
| $75,000–$100,000 | 17 (19%) |
| >$100,000 | 11 (13%) |
| **Marital Status** |  |
| Married | 17 (19%) |
| Unmarried | 71 (81%) |
|  |  |

|  |  |
| --- | --- |

**Supplementary Table 2.** Summary of polymorphisms associated with subjective well-being (SWB), neuroticism (N) and depressive symptoms (DS) from Okbay and colleagues^17^.

| **Phenotype** | **CHR** | **Position** | **SNP** | **Allele** | **Beta** | **Name on Chip** |
| --- | --- | --- | --- | --- | --- | --- |
| N | 3 | 34,582,993 | rs35688236 | A | 0.0213 | Na |
| SWB | 5 | 130,951,750 | rs3756290 | A | -0.0177 | rs3756290:130951750:A:G |
| SWB | 5 | 152,187,729 | rs4958581 | T | 0.0153 | rs4958581:152187729:T:C |
| *DS* | 5 | 164,483,794 | rs4481363 | A | 0.0140 | Na |
| *N* | 5 | 164,474,719 | rs4481363 | A | 0.0151 | Na |
| *DS* | 6 | 27,491,299 | rs4346787 | A | -0.0230 | rs6904596:27491299:G:A |
| *N* | 6 | 27,491,299 | rs6904596 | A | -0.0264 | rs6904596:27491299:G:A |
| N | 8 | 11,105,077 | rs2572431 | T | 0.0283 | rs2572431:11105077:C:T |
| N | 9 | 11,699,270 | rs10960103 | C | 0.0264 | rs10960103:11699270:C:G |
| N | 9 | 23,316,330 | rs2150462 | C | -0.0217 | Na |
| N | 11 | 113,364,803 | rs4938021 | T | 0.0233 | rs4938021:113364803:C:T |
| N | 11 | 10,253,183 | rs139237746 | T | -0.0204 | rs139237746:10253183:T:C |
| *N* | 11 | 47,663,049 | rs10838738 | A | 0.0178 | exm-rs10838738 |
| DS | 12 | 118,375,486 | rs7973260 | A | 0.0306 | rs79732609:33048667:A:C |
| *N* | 12 | 117,674,129 | rs10774909 | C | -0.0150 | Na |
| N | 15 | 78,033,735 | rs12903563 | T | 0.0198 | Na |
| N | 17 | 44,142,332 | rs193236081 | T | -0.0284 | 17:44142332:T:G |
| N | 17 | 2,574,821 | rs12938775 | A | -0.0202 | Na |
| N | 18 | 35,127,427 | rs1557341 | A | 0.0213 | rs1557341:35127427:A:C |
| N | 18 | 35,364,098 | rs12961969 | A | 0.0250 | rs12961969 |
| DS | 18 | 50,754,633 | rs62100776 | A | -0.0252 | Na |
| SWB | 20 | 47,701,024 | rs2075677 | A | 0.0175 | rs2075677:47701024:A:G |

Notes: Single nucleotide polymorphisms (SNPs) emerging at the genome-wide significance level (see underlined phenotype labels) as well as via proxy-phenotype analyses of subjective well-being loci with P < 1 × 10^−4^ (see italicized phenotype labels) from Okbay et al.^17^ are listed (see Table 1 in Okbay et al.). CHR: chromosome

**Supplementary Table 3.** Summary of inter-correlations among units of analysis

|  |  | **SHPS** | **Put volume** | **NAc volume** | **NAc RPE** | **Put RPE** |
| --- | --- | --- | --- | --- | --- | --- |
| **Reward learning** | Pearson r | -0.178 | -0.059 | 0.008 | -0.058 | 0.143 |
|  | p-value | 0.130 | 0.644 | 0.950 | 0.667 | 0.289 |
|  | N | 74 | 63 | 63 | 57 | 57 |
|  |  |  |  |  |  |  |
| **SHAPS** | Pearson r |  | 0.022 | -0.100 | -0.044 | 0.028 |
|  | p-value |  | 0.853 | 0.403 | 0.728 | 0.826 |
|  | N |  | 72 | 72 | 64 | 64 |
|  |  |  |  |  |  |  |
| **Bilateral Put volume** | Pearson r |  |  | .652** | 0.058 | 0.057 |
|  | p-value |  |  | 0.00001 | 0.650 | 0.657 |
|  | N |  |  | 73 | 63 | 63 |
|  |  |  |  |  |  |  |
| **Bilateral NAc volume** | Pearson r |  |  |  | 0.177 | 0.146 |
|  | p-value |  |  |  | 0.166 | 0.252 |
|  | N |  |  |  | 63 | 63 |
|  |  |  |  |  |  |  |
| **Bilateral NAc RPE** | Pearson r |  |  |  |  | .719** |
|  | p-value |  |  |  |  | 0.00001 |
|  | N |  |  |  |  | 65 |
|  |  |  |  |  |  |  |

Notes: SHAPS: *Reward learning* refers to stress-induced changes in reward learning. *RPE* refers to stress-induced changes in reward prediction error. For both the functional and the structural MRI, bilateral regions are considered. SHAPS: Snaith-Hamilton Pleasure Scale; NAc: nucleus accumbens; Put: putamen.
